# Association between academic pressure, *NR3C1* gene methylation, and anxiety symptoms among Chinese adolescents: a nested case-control study

**DOI:** 10.1186/s12888-023-04816-7

**Published:** 2023-05-30

**Authors:** Yilin Hua, Cuihong Huang, Yangfeng Guo, Xueying Du, Liling Guo, Wanxin Wang, Ciyong Lu, Lan Guo

**Affiliations:** 1 Department of Medical Statistics and Epidemiology, School of Public Health, Sun Yat-sen University, 74 Zhongshan Rd 2, 510080 Guangzhou, People’s Republic of China; 2 Guangdong Provincial Key Laboratory of Food, Nutrition and Health, Sun Yat-sen University, 510080 Guangzhou, People’s Republic of China; 3 Health Promotion Center for Primary and Secondary Schools, Guangzhou, People’s Republic of China

**Keywords:** Anxiety symptoms, Academic pressure, DNA methylation, Adolescents

## Abstract

**Background:**

Academic pressure is a prevalent stressor among Chinese adolescents and is often linked to anxiety symptoms, although the underlying mechanism remains unclear. This study aimed to investigate the association between *NR3C1* gene methylation, academic pressure, and anxiety symptoms among Chinese adolescents.

**Methods:**

This nested-case control study included 150 adolescents (boys: 38.7%; baseline age: 12–17 years) from a school-based longitudinal study of Chinese adolescents. Cases (n = 50) were defined as those with anxiety symptoms at both baseline and follow-up, while controls (n = 100) were randomly selected from those without anxiety symptoms at both timepoints. The cases and controls were 1:2 matched by age. Academic pressure, anxiety symptoms, and potential covariates were measured using a self-report questionnaire. Peripheral whole blood samples were collected from each participant for the detection of cortisol level (i.e., morning serum cortisol level) and DNA methylation. The methylation analysis included a total of 27 CpG units at the *NR3C1* promoter region.

**Results:**

The final adjusted models showed that students with heavy academic pressure at baseline were at a higher risk of anxiety symptoms at follow-up compared to those with mild academic pressure (*β* estimate: 6.24 [95% CI: 3.48 ~ 9.01]). After adjusting for covariates, the methylation level of one CpG unit (*NR3C1*-16 CpG10) in *NR3C1* differed significantly between cases and controls (F = 6.188, *P* = 0.014), and the difference remained significant after correction for multiple testing (*P* < 0.025). The adjusted regression models showed that moderate (*β* estimate = 0.010 [95% CI: 0.000 ~ 0.020], *P* = 0.046) and heavy (*β* estimate = 0.011 [95% CI: 0.001 ~ 0.020], *P* = 0.030) academic pressure were significantly associated with the methylation level of *NR3C1*-16 CpG 10. Further mediation analysis demonstrated that the association of academic pressure and anxiety symptoms was significantly mediated by the methylation of *NR3C1*-16 CpG 10 (*β* estimate for indirect effect = 0.11 [95% CI: 0.005 ~ 0.32]; indirect/total effect = 8.3%).

**Conclusion:**

The present study suggests that *NR3C1*-16 CpG 10 DNA methylation might be a potential mechanism that partially explains the lasting effects of academic pressure on subsequent anxiety symptoms among adolescents. Further studies with larger sample sizes are recommended to replicate this finding.

**Supplementary Information:**

The online version contains supplementary material available at 10.1186/s12888-023-04816-7.

## Background

Adolescence is a pivotal developmental period for physical maturation, social role changes, and the formation of the brain mechanisms of self-regulation during which the risk for mental health problems markedly increases [1]. Anxiety symptoms are among the most prevalent mental health problems among adolescents [2] and may generate a substantial societal burden due to impaired social functioning, decreased school attendance, or poor academic achievement [3–5]. Moreover, anxiety symptoms may serve as risk factors for persistent symptoms or full-blown anxiety disorders [6]. Previous studies suggest that children with persistent high and increasing levels of anxiety symptoms are five times more likely to be diagnosed with an anxiety disorder than those with low and stable anxiety symptoms [7]. A recent meta-analysis reported that the overall pooled prevalence rate of elevated anxiety symptoms among adolescents was 20.5% globally during the COVID-19 pandemic [8], with approximately 37.4% of Chinese adolescents reporting having anxiety symptoms [9]. These studies highlight the continued importance of identifying risk factors or potential mechanisms for adolescent anxiety symptoms from a public health perspective.

Although anxiety symptoms are multifactorial [10], compelling evidence suggests that stressful life events are significantly related to anxiety symptoms by disrupting the stress response system [11]. Academic pressure, referred to a student’s response to academic-related demands that exceed adaptive capabilities, is a common stressor among adolescents during their academic careers [12], particularly in China, where education and academic performance are highly valued [13]. Previous evidence suggests that academic pressure has become a serious social issue in China due to educational inequalities, and Chinese adolescents face immense academic pressure from the expectations of parents, teachers, and themselves to excel academically [14, 15]. Several epidemiological studies have demonstrated that academic pressure is positively associated with an increased risk of anxiety symptoms among adolescents [16, 17]. However, there is a paucity of research exploring the potential mechanisms underlying the effects of academic pressure on anxiety symptoms.

Evidence suggests that prolonged exposure to stressful life events may disrupt the stress response system [18]. The hypothalamus-pituitary-adrenal (HPA) axis is the central stress response system, and recent studies have reported that epigenetic modifications, such as DNA methylation changes, are thought to link stressful life events to susceptibility to anxiety symptoms by interfering with the HPA axis response and adaptation to stressors [19]. The epigenetic process of DNA methylation involves the addition of methyl groups to cytosine-guanine dinucleotides (CpGs) in gene promoters and regulatory regions, regulating gene transcription without changing the DNA sequence [20]. Genes such as FK506 binding protein 5 gene (*FKBP5*) and glucocorticoid receptor (GR) gene (*NR3C1*), which are related to the negative feedback mechanism of the HPA axis, have drawn increasing attention in the field of epigenetics and mental health. Several types of stressful life events have been frequently reported to alter the methylation of these genes [19, 21]. Although our prior works did not observe a significant association between *FKBP5* methylation and anxiety symptoms [22, 23], the *NR3C1* gene, which encodes GR and regulates the release of glucocorticoids in the HPA axis [24], also merits attention. Previous studies have demonstrated that hypermethylation of the *NR3C1* gene is associated with internalizing psychopathologies such as anxiety or depression [25, 26]. However, a study on combat veterans found that those with posttraumatic stress disorder (PTSD) had lower levels of methylation in the *NR3C1*-1 F promoter region, compared with those without PTSD [27]. The direction of methylation remains inconclusive, suggesting the need for further research. Moreover, despite traumatic life experiences, other common life stressors may also alter the *NR3C1* methylation level [28]. Consequently, it is speculated that DNA methylation of the *NR3C1* gene might also be altered by academic pressure, a common life stress among adolescents, and may play a vital role in the association between academic pressure and persistent anxiety symptoms. However, relatively few studies have evaluated these associations. Additionally, few related studies estimating the associations of *NR3C1* methylation with stress exposure and anxiety-related outcomes have accounted for other important confounders other than sex and age [29, 30], such as cortisol and body mass index (BMI), which have also been reported to be associated with anxiety and *NR3C1* DNA methylation [31, 32].

Therefore, we hypothesize that after controlling for confounders, heavy academic pressure may be associated with *NR3C1* methylation and anxiety symptoms, and the *NR3C1* methylation may play a mediating role in the link between academic pressure and anxiety symptoms. We tested these hypotheses with a nested case-control study design based on the Longitudinal Study of Adolescents’ Mental and Behavioral well-being Research (LSAMBR).

## Methods

### Study design and participants

**Fig. 1:**
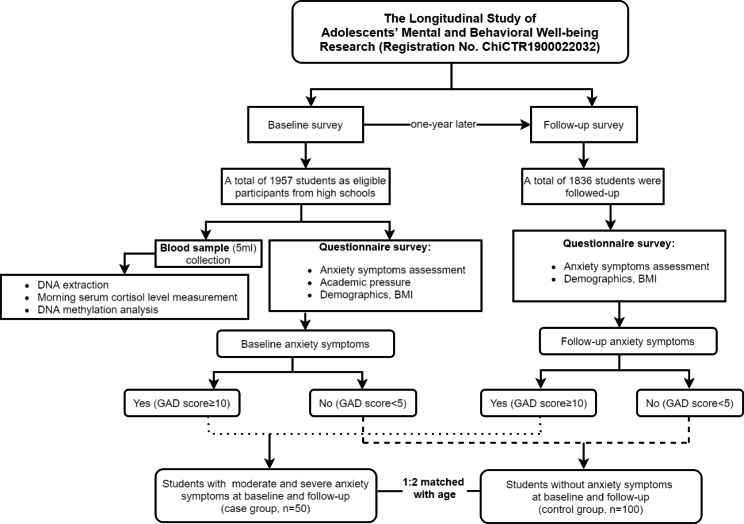
Flow chart of the nested case-control study

This study used a nested case-control study design based on the LSAMBR in Guangzhou, China (Registration No. ChiCTR1900022032). The LSAMBR is a prospective study, which has been carried out in six junior high schools and four senior high schools from 4 main districts of Guangzhou. Students in grade 7^th^ within the selected junior high schools and grade 10^th^ within the selected senior high schools were invited to participate voluntarily. A multistage, stratified cluster, random sampling method was used in the LSAMBER study. The study design has been described elsewhere in detail [33]. Briefly, between January and April in 2019, 1957 participants aged 11–18 years were interviewed at baseline (response rate: 99.03%), and followed up one year later (retention rate: 93.8%). A self-reported questionnaire was used to collect information, and the questionnaire was distributed in the classrooms with the absence of teachers and administered by our research assistants to reduce information bias. The inclusion criterion of the LSAMBR was the first-year students of the schools selected, while exclusion criteria included: (1) lack of fluency in Mandarin; (2) self-reported physician’s diagnosis of depressive disorder, severe psychiatric disorder, and/or alcohol or drug dependence disorder, which was assessed by asking the following question: “Have you ever been told by a doctor that you have been diagnosed with a depressive disorder/any severe psychiatric disorder/any alcohol or drug dependence disorder?”; and (3) inability to understand questionnaires or provide consent for themselves. In this 1:2 matched nested case-control study, students with moderate and severe anxiety symptoms at baseline and follow-up were treated as cases (n = 50), and those without anxiety symptoms at baseline and follow-up were randomly selected as the control group (n = 100). The case and control groups were matched for age (± 2 years) (Fig. 1). Ethical approval was obtained from the Sun Yat-sen University School of Public Health Institutional Review Board (Ethics Number: L2017060).

### Measures

#### Anxiety symptoms

Anxiety symptoms were evaluated using the Generalized Anxiety Disorder Scale-7 (GAD-7) in Chinese [34]. This scale has been validated and extensively utilized in Chinese studies with satisfactory psychometric properties [9], and the Cronbach’s alpha for baseline and follow-up assessments were 0.924 and 0.855, respectively. Participants were asked to report the frequency of 7 symptoms of anxiety on a 4-point scale ranging from “not at all = 0” to “nearly every day = 3”. The total score ranges from 0 to 21, with higher scores indicating more severe anxiety symptoms. In this study, participants with a baseline and follow-up GAD score of ≥ 10 were classified as the case group, representing those with persistently moderate to severe anxiety symptoms, while those with a GAD score < 5 at baseline and follow-up were considered as controls continuously without anxiety symptoms [34].

#### Academic pressure

Perceived academic pressure was assessed by asking students about their perception of academic pressure at school. The responses were categorized into “mild = 1”, “moderate = 2”, and “heavy = 3” [35].

#### Other information

Demographic information including age, sex (boy, girl), relationships with teachers (good, average, and poor), relationships with classmates (good, average, and poor), and living arrangement (living with others, living with a single parent, and living with both parents). BMI was calculated as an individual’s weight in kilograms divided by the square of height in meters.

#### Blood collection, DNA extraction, and morning serum cortisol level measurement

At baseline survey, peripheral whole blood samples (5 ml) were collected from each student using EDTA anticoagulant tubes and stored at − 80 °C freezer for further study. According to the manufacturer’s instructions, genomic DNA was extracted from peripheral blood using standard techniques by the DNA extraction kit (BioTeke Corporation, Beijing, China). DNA concentration was estimated at the wavelength of A260 nm by a NanoDrop 2000 C spectrophotometer (Thermo Scientific, Waltham, MA, USA).

Another 4-mL sample of whole blood was drawn from 7:00 to 10: 00 am to obtain serum. Morning serum cortisol levels were also measured using the competitive chemiluminescent microparticle immunoassay with the Abbott Architect i2000SR system (Abbott Laboratories, Abbott Park, IL).

#### DNA methylation analysis

A total of 42 CpG sites in the promoter region of the *NR3C1* region were selected as targets for methylation analysis. The sequences of CpG islands (Chr5: 143402787 ~ 143403213; Chr5: 143403353 ~ 143403714) were determined through the CpG Island Online Prediction website (http://www.ebi.ac.uk/Tools/seqstats/emboss_cpgplot/) based on the CpG island determination criteria (Observed/Expected ratio > 0.60; Length > 200; %GC > 50). Agena EpiDesigner software was used to design PCR primers for the target sequence, and two optimal primer design schemes (#10 and #16; details in **eMethods 1**) were selected. Bisulfite conversion of the DNA was performed using EZ DNA Methylation Kit (Zymo Research, Orange, USA). PCR amplification was performed using the following primers to obtain an amplification product with a T7 RNA polymerase promoter sequence: forward 5’-AGGAAGAGAGAGGTATTTAAGGGTATTTTTGGTGG-3’, reverse biotin-5’-CAGTAATACGACTCACTATAGGGAGAAGGCTCCTATAATCCCAACATTTTAAAAAACC-3’. In the in-vitro transcription system, the amplification product was transcribed into RNA fragments by T7 RNA polymerase, followed by base-specific cleavage using RNase A. The resulting small RNA fragments carrying CpG sites differed in their nucleotide composition depending on the bisulfite treatment. DNA methylation levels were quantified using the SEQUENOM MassARRAY EpiTYPER platform, which produced results for each unit of analysis, called a CpG unit, containing an individual CpG site or an aggregate of several CpG sites whose positions were relatively close [36]. Further quality control was conducted, including excluding CpG units with less than 80% of available methylation data to ensure that spurious data were not analyzed [37]. Besides, significantly deviating data points were also excluded [38]. A total of 27 CpG units, encompassing 42 CpG sites were ultimately qualified for analysis (The locations of the CpG units were presented in Table S1).

### Statistical analysis

First, data normality was assessed by visual inspection of histograms and the Shapiro-Wilk test. Descriptive statistics were stratified by students with persistent anxiety symptoms (cases) and those without anxiety symptoms (controls) to depict sample characteristics. Continuous and categorical data were presented as proportions, means (standard deviation, SD), and medians (interquartile range, IQR) as appropriate. To estimate the difference between cases and controls in sample characteristics and DNA methylation levels of the *NR3C1* gene, univariable analysis (using Chi-square tests, Wilcoxon rank tests, and *t*-tests) was performed. The false discovery rate (FDR) was calculated to address multiple hypothesis testing and potential type I errors for the differences between cases and controls in the DNA methylation levels of the *NR3C1* gene. The FDR-adjusted *P* was presented by “*q*”, and the results were considered as nominally significant if *q* < 0.10. Second, multivariable analysis of covariance (MANCOVA) was conducted to explore the differences between cases and controls in *NR3C1* DNA methylation levels, adjusting for variables that were significant with *P* < 0.10 in the univariable analyses or widely reported to be associated with anxiety symptoms [31, 32, 39–41]. Statistical significance was adjusted for multiple comparisons of the number of observed statistically significant CpG units in univariable analysis using the Bonferroni method. Third, univariable and multivariable linear regression models were conducted to estimate the associations between academic pressure and *NR3C1* DNA methylation levels. A mediation analysis was performed to estimate the role of *NR3C1* DNA methylation in the association between academic pressure and subsequent anxiety symptoms using the SPSS implementation of PROCESS 37. All statistical analyses were conducted using Stata 16.0 SE (StataCorp, Houston, Texas, USA) and SPSS (SPSS 25.0 (IBM Corp, Armonk, NY, USA), with all statistical tests being two-sided.

## Results

### Baseline sample characteristics

The sample characteristics at baseline are shown in Table 1. The case group consisted of 50 students with persistent anxiety symptoms, and the control group consisted of 100 students who continuously did not exhibit anxiety symptoms. The two groups were age-matched (*P* = 0.438), with a median age of 13.0 years (interquartile range: 12.8 to 14.3) for cases and 13.0 years (interquartile range: 12.3 to 15.0) for controls. In the case group, 70.0% were girls, which was higher than the 57.0% in the control group, although the difference was not statistically significant (*P* = 0.155). There were no statistical differences between the cases (BMI: 18.7 [17.1 to 20.3] kg/m^2^; cortisol: 207.9 [152.3 to 312.3] nmol/L) and controls (BMI: 19.1 [17.6 to 21.6] kg/m^2^; cortisol: 213.0 [141.1 to 329.5] nmol/L) in the distributions of BMI and morning cortisol level (BMI: *P* = 0.063; cortisol: *P* = 0.541). The proportion of students reporting poor classmate relations was higher in the case group (12.0%) compared to the control group (7.1%), with a statistical difference (*P* < 0.001). There were no statistical differences between cases and controls in the distributions of teacher-classmate relations and living arrangement (all *P* > 0.05). Importantly, the case group had a higher proportion of students reporting heavy academic pressure (72.0%) compared to the control group (36.0%), with a statistically significant difference (*P* < 0.001).

**Table 1:** Sample characteristics between cases and control group

Variable	Non-anxiety symptoms group (n, %)	Persistent anxiety symptoms group (n, %)	*P-* value*
**Total**	100 (100)	50 (100)	
**Age**, median (interquartile range), year	13.0 (12.3 to 15.0)	13.0 (12.8 to 14.3)	0.438
**Sex**			
Boy	43 (43.0)	15 (30.0)	0.155
Girl	57 (57.0)	35 (70.0)	
**BMI**, kg/m^2^	18.7 (17.1 to 20.3)	19.1 (17.6 to 21.6)	0.063
**Morning serum total cortisol**, median (interquartile range), nmol/L	207.9 (152.3 to 312.3)	213.0 (141.1 to 329.5)	0.845
**Classmate relations**			
Poor	7 (7.1)	6 (12.0)	< 0.001
Average	13 (13.1)	20 (40.0)	
Good	79 (79.8)	24 (48.0)	
**Teacher-classmate relations**			
Poor	1 (1.0)	1 (2.0)	0.165
Average	24 (24.0)	19 (38.0)	
Good	75 (75.0)	30 (60.0)	
**Living arrangement**			
Living with others	6 (6.0)	7 (14.0)	0.225
Living with a single parent	14 (14.0)	8 (16.0)	
Living with both parents	80 (80.0)	35 (70.0)	
**Academic pressure**			
Mild	25 (25.0)	1 (2.0)	< 0.001
Moderate	39 (39.0)	13 (26.0)	
Heavy	36 (36.0)	36 (72.0)	
**GAD scores at baseline**, mean SD	2.8 (1.9)	15.1 (3.5)	< 0.001
**GAD scores at follow-up**, mean SD	2.6 (1.9)	15.7 (3.7)	< 0.001

Abbreviations: Generalized Anxiety Disorder Scale-7, GAD-7.

*: The chi-square tests were used for categorical variables, the Wilcoxon rank tests were used for age, BMI, and morning serum total cortisol data, and the *t*-tests were used for GAD scores data

### Association between baseline academic pressure and anxiety symptoms

As shown in Table S2, compared with those with mild academic pressure, adolescents with moderate (OR = 8.33, 95% CI = 1.03 ~ 67.71) and heavy academic pressure (OR = 25.00, 95% CI = 3.21 ~ 194.48) were at a higher risk of being in persistent anxiety symptoms status. After adjusting for age, sex, BMI, cortisol, living arrangement, classmate relations, and teacher-classmate relations, these associations remained significant. Moreover, univariable linear regression models showed that moderate (*β* estimate = 3.44, 95% CI = 0.68 ~ 6.21) and heavy (*β* estimate = 8.25, 95% CI = 5.62 ~ 10.89) academic pressure were positively associated with anxiety symptoms score at follow-up. After adjusting for the above-mentioned variables as well as baseline anxiety symptoms scores, the association between heavy academic pressure and anxiety symptoms score at follow-up remained significant (*β* estimate = 6.24, 95% CI = 3.48 ~ 9.01).

### Group differences in the *NR3C1* DNA methylation level

As shown in Table 2, among the 24 CpG units in the promoter region of the *NR3C1* gene detected in this study, the differences in methylation levels between cases and controls were only significant at *NR3C1*-16 CpG 9 (cases: 0.04 [0.02 to 0.10] vs. controls: 0.03 [0.00 to 0.07], *P* = 0.022) and *NR3C1*-16 CpG 10 (cases: 0.03 [0.02 to 0.05] vs. controls: 0.02 [0.01 to 0.03], *P* = 0.002). After further correction for multiple testing, the difference in methylation levels between cases and controls at *NR3C1*-16 CpG10 remained statistically significant (*q* < 0.10).

**Table 2:** NR3C1 DNA methylation levels between cases and control group

CpG unit	Non-anxiety symptoms group^*^	Persistent anxiety symptoms group^*^	*P-*value^a^	*q-*value^*#*^
*NR3C1*-10 CpG 1	0.06 (0.05 to 0.07)	0.06 (0.05 to 0.07)	0.647	0.855
*NR3C1*-10 CpG 2	0.03 (0.01 to 0.05)	0.03 (0.01 to 0.05)	0.733	0.855
*NR3C1*-10 CpG 3.4.5	0.03 (0.02 to 0.04)	0.03 (0.02 to 0.05)	0.818	0.855
*NR3C1*-10 CpG 6.7.8	0.06 (0.04 to 0.07)	0.06 (0.04 to 0.08)	0.574	0.855
*NR3C1*-10 CpG 9.10.11	0.04 (0.02 to 0.05)	0.04 (0.02 to 0.05)	0.821	0.855
*NR3C1*-10 CpG 12.13	0.07 (0.05 to 0.08)	0.08 (0.05 to 0.09)	0.148	0.765
*NR3C1*-10 CpG 15.16.17	0.02 (0.01 to 0.03)	0.02 (0.01 to 0.02)	0.728	0.855
*NR3C1*-10 CpG 18.19.20	0.13 (0.10 to 0.17)	0.13 (0.09 to 0.18)	0.734	0.855
*NR3C1*-10 CpG 21	0.05 (0.02 to 0.12)	0.06 (0.03 to 0.11)	0.714	0.855
*NR3C1*-10 CpG 22.23.24	0.03 (0.02 to 0.05)	0.04 (0.02 to 0.05)	0.735	0.855
*NR3C1*-10 CpG 25.26	0.02 (0.00 to 0.04)	0.01 (0.00 to 0.04)	0.690	0.855
*NR3C1*-10 CpG 27.28	0.02 (0.00 to 0.04)	0.01 (0.00 to 0.04)	0.690	0.855
*NR3C1*-10 CpG 29	0.12 (0.10 to 0.14)	0.12 (0.10 to 0.14)	0.368	0.855
*NR3C1*-16 CpG 1	0.00 (0.00 to 0.04)	0.00 (0.00 to 0.03)	0.609	0.855
*NR3C1*-16 CpG 2	0.00 (0.00 to 0.00)	0.05 (0.00 to 0.78)	0.079	0.658
*NR3C1*-16 CpG 3	0.03 (0.01 to 0.08)	0.03 (0.00 to 0.06)	0.344	0.855
*NR3C1*-16 CpG 4	0.02 (0.00 to 0.05)	0.03 (0.00 to 0.05)	0.153	0.765
*NR3C1*-16 CpG 5	0.07 (0.03 to 0.13)	0.09 (0.04 to 0.13)	0.461	0.855
*NR3C1*-16 CpG 6.7	0.02 (0.01 to 0.04)	0.03 (0.02 to 0.04)	0.190	0.792
*NR3C1*-16 CpG 8	0.01 (0.00 to 0.03)	0.00 (0.00 to 0.03)	0.561	0.855
*NR3C1*-16 CpG 9	0.03 (0.00 to 0.07)	0.04 (0.02 to 0.10)	**0.022**	0.188
*NR3C1*-16 CpG 10	0.02 (0.01 to 0.03)	0.03 (0.02 to 0.05)	**0.002**	**0.050**
*NR3C1*-16 CpG 11	0.04 (0.02 to 0.05)	0.04 (0.02 to 0.05)	0.946	0.946
*NR3C1*-16 CpG 12	0.10 (0.05 to 0.16)	0.12 (0.03 to 0.22)	0.419	0.855
*NR3C1*-16 CpG 13	0.06 (0.02 to 0.13)	0.07 (0.01 to 0.16)	0.813	0.855

*: DNA methylation data were described as medians (interquartile range, IQR).

a: The Wilcoxon rank tests were used to compare the differences in methylation levels between cases and controls. The *P*-values less than 0.05 were shown in bold type

^#^: “*q* value” indicates the FDR-adjusted *P* value, and the results were considered as nominally significant when *q* < 0.10. The *q*-values less than 0.10 were shown in bold type

Table 3 shows the results of the multivariable analysis of covariance (MANCOVA) of DNA methylation levels at the two significant *NR3C1* CpG units between cases and controls. After adjusting for age, sex, BMI, cortisol, living arrangement, classmate relations, and teacher-classmate relations, Model 1 showed that the *NR3C1*-16 CpG10 methylation level differed significantly between students with persistent anxiety symptoms (cases) and those without anxiety symptoms (controls) (F = 6.188, *P* = 0.014, ${{\eta }_{p}}^{2}=0.045)$, even after correcting for multiple testing using the Bonferroni method (*P*<0.025). The *NR3C1*-16 CpG10 methylation level in the cases group was higher than that in the control group. After adjusting for the variables in Model 1 plus academic pressure, the difference between cases and controls in the DNA methylation level of *NR3C1*-16 CpG 10 remained significant (F=4.512, *P*=0.036, ${{\eta }_{p}}^{2}=0.034).$ However, after correcting for multiple testing, no significant difference was observed (*P*>0.025).

**Table 3:** Multivariate analysis of covariance of NR3C1 methylation levels between cases and control group

CpG unit^#^	Group					
	Model 1 (Statistics: Wilks λ = 0.949, F (2, 130) = 3.498, *P* = 0.033)			Model 2 (Statistics: Wilks λ = 0.960, F (2, 128) = 2.690, *P* = 0.072)		
	**F**	***P*** ^*^	${{\varvec{\eta }}_{\varvec{p}}}^{2}$	**F**	***P*** ^*^	${{\varvec{\eta }}_{\varvec{p}}}^{2}$
*NR3C1-*16 CpG 9	1.208	0.274	0.009	1.091	0.298	0.008
*NR3C1*-16 CpG 10	6.188	0.014	0.045	4.512	0.036	0.034

^#^: Only the *NR3C1* CpG units observed with a significant association of anxiety symptoms in the unadjusted model were reported here

Model 1: MANCOVA demonstrated significant between-group difference after controlling for age, sex, BMI, cortisol, living arrangement, classmate relations, and teacher-classmate relations

Model 2: MANCOVA demonstrated significant between-group difference after controlling for age, sex, BMI, cortisol, living arrangement, classmate relations, teacher-classmate relations, and academic pressure

*: Statistical significance was set at *P* < 0.025 after Bonferroni correction for 2 CpG sites

### Associations between academic pressure, *NR3C1* DNA methylation level, and anxiety symptoms

Table 4 presented that compared to mild academic pressure, moderate and heavy academic pressure were positively associated with the methylation level of *NR3C1*-16 CpG 10 (average academic pressure: *β* estimate = 0.012, 95% CI = 0.002 ~ 0.022, *P* = 0.014, Model 1; heavy academic pressure: *β* estimate = 0.015, 95% CI = 0.006 ~ 0.024, *P* = 0.001, Model 1) before adjusting for other variables. After adjusting for age, sex, BMI, cortisol, living arrangement, classmate relations, and teacher-classmate relations, the positive associations of moderate academic pressure (*β* estimate = 0.010, 95% CI = 0.000 ~ 0.020, *P* = 0.046; Model 2) and heavy academic pressure (*β* estimate = 0.011, 95% CI = 0.001 ~ 0.020, *P* = 0.030; Model 2) with *NR3C1*-16 CpG 10 methylation level remained significant.

**Table 4:** Associations between academic pressure and NR3C1 DNA methylation level

CpG unit	Baseline academic pressure (Ref.= mild academic pressure)							
	Moderate academic pressure				Heavy academic pressure			
	Model 1		Model 2		Model 1		Model 2	
	***β*** **estimate (95% CI)**	***P*** **-value** ^*^	***β*** **estimate (95% CI)**	***P*** **-value** ^*^	***β*** **estimate (95% CI)**	***P*** **-value** ^*^	***β*** **estimate (95% CI)**	***P*** **-value** ^*^
NR3C1-10 CpG 1	0.007 (-0.003 ~ 0.016)	0.158	0.008 (-0.001 ~ 0.018)	0.080	0.000 (-0.009 ~ 0.009)	0.977	0.003 (-0.006 ~ 0.013)	0.488
NR3C1-10 CpG 2	0.008 (-0.004 ~ 0.021)	0.197	0.009 (-0.003 ~ 0.022)	0.145	0.011 (-0.002 ~ 0.023)	0.098	-0.010 (-0.023 ~ 0.003)	0.122
NR3C1-10 CpG 3.4.5	0.009 (-0.002 ~ 0.020)	0.111	0.009 (-0.002 ~ 0.021)	0.105	0.006 (-0.005 ~ 0.016)	0.280	0.006 (-0.005 ~ 0.018)	0.277
NR3C1-10 CpG 6.7.8	0.008 (-0.004 ~ 0.021)	0.196	0.009 (-0.004 ~ 0.021)	0.179	0.006 (-0.006 ~ 0.018)	0.296	0.010 (-0.003 ~ 0.022)	0.141
NR3C1-10 CpG 9.10.11	0.006 (-0.006 ~ 0.018)	0.337	0.008 (-0.005 ~ 0.020)	0.215	0.003 (-0.009 ~ 0.015)	0.623	-0.001 (-0.013 ~ 0.011)	0.866
NR3C1-10 CpG 12.13	0.003 (-0.010 ~ 0.015)	0.697	0.001 (-0.012 ~ 0.013)	0.922	0.004 (-0.008 ~ 0.016)	0.468	0.001 (-0.012 ~ 0.014)	0.846
NR3C1-10 CpG 14	NA		NA		NA		NA	
NR3C1-10 CpG 15.16.17	0.004 (-0.006 ~ 0.015)	0.653	0.005 (-0.006 ~ 0.015)	0.404	-0.003 (-0.012 ~ 0.007)	0.597	0.002 (-0.009 ~ 0.013)	0.723
NR3C1-10 CpG 18.19.20	0.031 (0.002 ~ 0.060)	**0.035**	0.032 (0.002 ~ 0.062)	**0.035**	0.007 (-0.020 ~ 0.035)	0.599	0.004 (-0.026 ~ 0.034)	0.798
NR3C1-10 CpG 21	-0.001 (-0.048 ~ 0.046)	0.968	-0.004 (-0.051 ~ 0.044)	0.878	-0.018 (-0.063 ~ 0.026)	0.417	-0.032 (-0.080 ~ 0.016)	0.190
NR3C1-10 CpG 22.23.24	0.008 (-0.017 ~ 0.034)	0.516	0.011 (-0.015 ~ 0.037)	0.405	0.000 (-0.024 ~ 0.025)	0.972	-0.007 (-0.033 ~ 0.019)	0.609
NR3C1-10 CpG 25.26	0.005 (-0.011 ~ 0.021)	0.554	0.001 (-0.015 ~ 0.017)	0.906	0.002 (-0.013 ~ 0.017)	0.797	0.003 (-0.013 ~ 0.019)	0.730
NR3C1-10 CpG 27.28	0.005 (-0.011 ~ 0.021)	0.554	0.001 (-0.015 ~ 0.017)	0.906	0.002 (-0.013 ~ 0.017)	0.797	0.003 (-0.013 ~ 0.019)	0.730
NR3C1-10 CpG 29	-0.007 (-0.024 ~ 0.011)	0.458	-0.006 (-0.023 ~ 0.012)	0.543	0.004 (-0.013 ~ 0.021)	0.674	0.008 (-0.009 ~ 0.026)	0.353
NR3C1-16 CpG 1	-0.003 (-0.021 ~ 0.015)	0.745	-0.005 (-0.024 ~ 0.013)	0.565	-0.009 (-0.026 ~ 0.009)	0.331	-0.007 (-0.026 ~ 0.011)	0.435
NR3C1-16 CpG 2	-0.124 (-0.315 ~ 0.066)	0.201	-0.115 (-0.339 ~ 0.108)	0.313	0.033 (-0.181 ~ 0.247)	0.763	0.239 (-0.024 ~ 0.502)	0.075
NR3C1-16 CpG 3	-0.003 (-0.046 ~ 0.040)	0.898	0.001 (-0.042 ~ 0.043)	0.982	-0.001 (-0.042 ~ 0.040)	0.963	0.010 (-0.032 ~ 0.053)	0.636
NR3C1-16 CpG 4	0.011 (-0.003 ~ 0.026)	0.113	0.008 (-0.007 ~ 0.022)	0.304	0.008 (-0.005 ~ 0.022)	0.229	0.005 (-0.009 ~ 0.020)	0.470
NR3C1-16 CpG 5	-0.005 (-0.042 ~ 0.033)	0.808	-0.007 (-0.045 ~ 0.031)	0.711	0.009 (-0.027 ~ 0.044)	0.623	0.011 (-0.027 ~ 0.049)	0.570
NR3C1-16 CpG 6.7	-0.011 (-0.021~-0.001)	**0.023**	-0.012 (-0.022~-0.003)	**0.012**	-0.008 (-0.017 ~ 0.001)	0.098	-0.006 (-0.015 ~ 0.004)	0.257
NR3C1-16 CpG 8	0.001 (-0.012 ~ 0.014)	0.904	-0.001 (-0.014 ~ 0.012)	0.835	-0.004 (-0.017 ~ 0.008)	0.515	-0.008 (-0.021 ~ 0.005)	0.226
NR3C1-16 CpG 9	0.019 (-0.019 ~ 0.056)	0.325	0.012 (-0.027 ~ 0.051)	0.547	0.016 (-0.020 ~ 0.051)	0.386	0.007 (-0.032 ~ 0.045)	0.742
NR3C1-16 CpG 10	0.012 (0.002 ~ 0.022)	**0.014**	0.010 (0.000 ~ 0.020)	**0.046**	0.015 (0.006 ~ 0.024)	**0.001**	0.011 (0.001 ~ 0.020)	**0.030**
NR3C1-16 CpG 11	0.006 (-0.005 ~ 0.016)	0.290	0.005 (-0.005 ~ 0.016)	0.310	0.004 (-0.005 ~ 0.014)	0.372	0.006 (-0.005 ~ 0.016)	0.272
NR3C1-16 CpG 12	0.036 (-0.022 ~ 0.093)	0.228	0.034 (-0.026 ~ 0.094)	0.272	0.022 (-0.033 ~ 0.077)	0.436	0.021 (-0.040 ~ 0.081)	0.501
NR3C1-16 CpG 13	0.002 (-0.055 ~ 0.059)	0.951	0.017 (-0.042 ~ 0.077)	0.567	-0.021 (-0.076 ~ 0.033)	0.445	-0.007 (-0.066 ~ 0.053)	0.829

Abbreviations: 95% CI, 95% confidence interval; NA, not applicable or not available

Model 1: unadjusted models

Model 2: adjusted for age, sex, BMI, cortisol, living arrangement, classmate relations, and teacher-classmate relations

*: The *P*-values less than 0.05 were shown in bold type

As shown in Table S3, the mediation models further presented that without adjusting for other variables only *NR3C1*-16 CpG10 DNA methylation mediated the associations between academic pressure and anxiety symptoms (*β* estimate = 0.17, 95% CI = 0.04 ~ 0.40; indirect/total effect = 12.8%). After adjusting for age, sex, BMI, cortisol, living, classmate relations, and teacher-classmate relations, the indirect effect for *NR3C1*-16 CpG10 methylation remained significant (*β* estimate = 0.11, 95% CI = 0.005 ~ 0.32; indirect/total effect = 8.3%).

## Discussion

This study provides evidence that a higher level of academic pressure was positively associated with anxiety symptoms among adolescents, even after adjusting for age, sex, BMI, cortisol, living arrangement, classmate relations, and teacher-classmate relations. Similarly, Zhu et al. found a positive correlation between academic pressure and anxiety symptoms among Chinese adolescents [16], Karki et al. reported a significant association between perceived academic pressure and anxiety symptoms among high school students in Nepal [42], and Trevethan et al. observed a longitudinal direct effect of academic pressure on changes in symptoms of generalized anxiety and panic [43]. According to Lazarus’s cognitive appraisal theory, stress is seen as a relation between individuals and their environment [44]. One possible explanation for these findings is that individual academic pressure may disrupt the development of an individual’s stress response system [18], which could increase susceptibility to anxiety symptoms by interfering with coping and adaptation to stressors [19]. Another explanation may be that non-supportive reactions to children’s academic pressure or performance from family or society (e.g., disciplining the child, poor family bonding, or communication) may exacerbate children’s negative emotional responses to academic pressure and elevate the risk of developing anxiety symptoms [45].

The *NR3C1* gene encoding GR is reported to be involved in the regulation of the stress response system by affecting the negative feedback mechanism of the HPA axis [24]. Previous studies have indicated that the DNA methylation status of the *NR3C1* gene is related to internalizing mental health problems, including anxiety symptoms [25, 26]. In this study, we estimated the *NR3C1* DNA methylation level between groups of students with persistent anxiety symptoms (cases) and controls without anxiety symptoms. One significant finding was that, after adjusting for potential confounders, including age, sex, BMI, cortisol, living arrangement, classmate relations, and teacher-classmate relations, the methylation level at one CpG unit (*NR3C1*-16 CpG10) at the *NR3C1* promoter region was significantly higher in the cases compared to controls. This finding remained statistically significant even after correction for multiple testing. Similarly, Wang et al. reported significantly increased methylation at the *NR3C1* promoter region among patients with generalized anxiety disorder [46]. A prospective study also found that methylation in one region of the *NR3C1* promoter may predict the development of internalizing problems, including anxiety symptoms [47]. Another study on Swedish adolescents reported a significant association between *NR3C1* exon 1 F hypermethylation and internalizing symptoms [26]. However, Tyrka et al. reported a negative association between *NR3C1* promoter methylation and anxiety disorders among adults [48]. The heterogeneity of the study population, the differences in the location of CpG sites, and the diversity of measurements may explain some of the inconsistencies in findings, highlighting the complexity of the epigenetic regulatory mechanism of *NR3C1* in relation to adverse psychological outcomes. Notably, when we controlled for the potential confounders mentioned above, as well as academic pressure, the significant difference in the methylation level of this CpG unit between cases and controls was no longer observed after correction for multiple testing. This finding suggests that academic pressure may be an important factor in the pathway to anxiety symptoms through the alteration of *NR3C1* methylation.

Academic pressure is one of the most common psychological stressors experienced by Chinese adolescents, arising not only from parents but also from peers and teachers, and has been associated with adverse psychological outcomes [49]. The dysfunction of the HPA axis has been identified as a vital mechanism by which stressful events can lead to these psychological outcomes[11]. In particular, academic pressure has been associated with acute or chronic alterations in HPA axis function, including changes in cortisol levels [50, 51]. Previous studies have shown that chronic examination stress is associated with an altered cortisol awakening response [52] and higher levels of hair cortisol concentration [51]. However, the current study did not observe a significant difference in the morning serum cortisol levels between adolescents with and without anxiety symptoms. In this study the morning serum cortisol was only measured once, reflecting the acute cortisol level, which may partially explain the inconsistency of findings with previous studies. Epigenetic modifications, specifically DNA methylation, have been proposed as a potential mechanism for stress-induced HPA axis dysfunction and as a biomarker for early identification and treatment of disease [53]. Although childhood trauma and prenatal stress have been associated with alterations in the methylation of the *NR3C1* gene [19], fewer studies have focused on more common daily life stressors, such as academic pressure [28]. It remained unclear whether academic pressure, a less acute but potentially more prevalent stressor among adolescents, can similarly alter the *NR3C1* methylation levels. The final adjusted multivariable linear models showed that moderate academic pressure was associated with altered methylation levels at three CpG units (*NR3C1*-10 CpG18.19.20, *NR3C1*-16 CpG6.7, and *NR3C1*-16 CpG10) of *NR3C1*, while heavy academic pressure was positively associated with the methylation of one CpG unit (i.e., *NR3C1*-16 CpG10). To the best of our knowledge, this is the first study to explore the association between academic pressure and *NR3C1* methylation. Our findings suggest that increased methylation of *NR3C1*-16 CpG10 was associated with both persistent anxiety symptoms and academic pressure. Further mediation analysis suggested that the impact of academic pressure on persistent anxiety symptoms might be partially mediated by the methylation of this CpG unit, even after adjusting for potential confounders. Methylation of *NR3C1* induced by academic pressure may result in aberrant expression of glucocorticoid receptors, disruption of the negative feedback of the HPA axis, and chronic or abnormal production of stress-related hormones, which may affect cognition and increase the susceptibility of anxiety symptoms [54]. Although epigenetic modifications are often reversible, DNA methylation is a relatively stable modification that can persistently interfere with gene expression and lead to adverse health outcomes (Moore et al., 2013). Therefore, our finding suggests that academic pressure may have lasting effects on the methylation of *NR3C1* and the susceptibility to anxiety symptoms, highlighting the importance of addressing academic pressure as a potential source of psychological stress in adolescents.

There are some limitations that warrant attention. First, although previous studies have utilized similar sample sizes to investigate the association of DNA methylation with stressful life events or negative psychological outcomes [25, 46], the sample size of our study is relatively small, which may imply insufficient statistical power. Nevertheless, we utilized a prospective nested case-control study design and rigorous criteria to include cases with persistent anxiety symptoms, which may have improved the research efficiency. Second, the effect size of one CpG unit within one gene could be relatively small, and some CpG units could not withstand correction for multiple testing. Additionally, we did not investigate the combined effect of *NR3C1* and other stress-related genes (e.g. *FKBP5*) in this study. Previous research has also shown that analyzing individual CpG sites can undermine the effects [55]. Therefore, we would like to expand our sample size and explore the joint effects of CpG sites of *NR3C1* and other related genes in future studies. Third, the DNA methylation assayed in this study was limited to the promoter region of *NR3C1*, and we would like to include a wider range of CpG sites across the gene in the future. Fourth, the biological sample assayed in this study was peripheral blood, and we cannot infer whether the methylation was consistent with that in the brain, the target organ. Although DNA methylation may vary between tissues [56], increasing evidence has suggested that blood-based measures as biomarkers can predict stress-related conditions in the brain [57]. Finally, the academic pressure was not measured by a complete scale due to questionnaire length limitations, which suggests a lack of evaluation of the psychometric properties for this measure in our study.

## Conclusion

In conclusion, this study indicates that elevated academic pressure and the consequent alterations in *NR3C1* methylation may serve as potential markers for identifying students with persistent anxiety symptoms. Our novel finding of a significant association between academic pressure and *NR3C1* methylation level change highlights the potential role of DNA methylation in anxiety etiology. Additionally, further mediation analysis shows that academic pressure’s lasting impact on anxiety symptoms may occur through altering the DNA methylation status of the *NR3C1-*16 CpG10 site. Although our study offers valuable insights into the relationship between DNA methylation and anxiety symptoms, more extensive studies are necessary to validate our results.

## Electronic supplementary material

Below is the link to the electronic supplementary material.

Supplementary Material 1: eMethods 1. The selection of CpG units in the study. Supplemental Table S1. The location of each CpG unit in the NR3C1 gene. Supplementary Table S2. The association of baseline academic pressure with subsequent anxiety symptoms. Supplementary Table S3. The mediating effect of NR3C1 methylation in the association of academic pressure with anxiety symptoms

## Data Availability

The datasets used and/or analyzed during the current study are available from the corresponding author on reasonable request.

## List of abbreviations

HPA axis: hypothalamus-pituitary-adrenal axis
GR: glucocorticoid receptors
*FKBP5*: FK506 binding protein 5 gene
*NR3C1*: glucocorticoid receptors gene
GAD-7: Generalized Anxiety Disorder Scale-7
