# Supplementary material for: Association between academic pressure, NR3C1 gene methylation, and anxiety symptoms among Chinese adolescents: a nested case-control study
Source: BMC Psychiatry. 2023 May 30;23:376. doi: 10.1186/s12888-023-04816-7 (PMC10227793; doi:10.1186/s12888-023-04816-7)
Supplement: Supplementary file 1 — Supplementary Material 1: eMethods 1. The selection of CpG units in the study. Supplemental Table S1. The location of each CpG unit in the NR3C1 gene. Supplementary Table S2. The association of baseline academic pressure with subsequent anxiety symptoms. Supplementary Table S3. The mediating effect of NR3C1 methylation in the association of academic pressure with anxiety symptoms [file 12888_2023_4816_MOESM1_ESM.docx]

**Supplement**

**eMethods 1.** The selection of CpG units in the study.

**Table S1.** The location of each CpG unit in the *NR3C1* gene.

**Table S2.** The association of baseline academic pressure with subsequent anxiety symptoms.

**Table S3.** The mediating effect of *NR3C1* methylation in the association of academic pressure with anxiety symptoms.

**eMethods 1. The selection of CpG units in the study.**

There is a total of 30 CpG sites in the DNA fragment corresponding to primer #10; only 29 of them are covered in our study.

**Primer #10**

Forward PCR primer:

5’ - aggaagagagTATAGATTATGATTTTTGTGATTTTGGG - 3’ (lower case letters indicate T7 primers)

Reverse PCR primer:

5’- cagtaatacgactcactatagggagaaggctACTTACTAAAAACCTACTTCTTTCCTTT - 3’ (lowercase letters indicate T7 primers)

There is a total of 13 CpG sites in the DNA fragment corresponding to primer #16; only 13 of them are covered in our study.

**Primer #16**

Forward PCR primer:

5’ - aggaagagagGAGTTGGATTTTTTTGTATTTTTTT - 3’ (lower case letters indicate T7 primers)

Reverse PCR primer:

5’ - cagtaatacgactcactatagggagaaggctAATTCCCTTCCCCTTAAACTAAA - 3’

(lowercase letters indicate T7 primers)

| **Table S1.** The location of each CpG unit in the *NR3C1* gene. | |
| --- | --- |
| **CpG unit** | **Location (chr5)** |
| *NR3C1*-10 CpG 1 | 143402871 |
| *NR3C1*-10 CpG 2 | 143402889 |
| *NR3C1*-10 CpG 3.4.5 | 143402908 |
|  | 143402912 |
|  | 143402922 |
| *NR3C1*-10 CpG 6.7.8 | 143402944 |
|  | 143402948 |
|  | 143402952 |
| *NR3C1*-10 CpG 9.10.11 | 143402977 |
|  | 143402980 |
|  | 143402982 |
| *NR3C1*-10 CpG 12.13 | 143402994 |
|  | 143402997 |
| *NR3C1*-10 CpG 14 | 143403026 |
| *NR3C1*-10 CpG 15.16.17 | 143403038 |
|  | 143403040 |
|  | 143403045 |
| *NR3C1*-10 CpG 18.19.20 | 143403052 |
|  | 143403056 |
|  | 143403059 |
| *NR3C1*-10 CpG 21 | 143403083 |
| *NR3C1*-10 CpG 22.23.24 | 143403091 |
|  | 143403097 |
|  | 143403099 |
| *NR3C1*-10 CpG 25.26 | 143403122 |
|  | 143403124 |
| *NR3C1*-10 CpG 27.28 | 143403128 |
|  | 143403130 |
| *NR3C1*-10 CpG 29 | 143403144 |
| *NR3C1*-10 CpG 30 | 143403171 |
| *NR3C1*-16 CpG 1 | 286806106 |
| *NR3C1*-16 CpG 2 | 286806132 |
| *NR3C1*-16 CpG 3 | 286806151 |
| *NR3C1*-16 CpG 4 | 286806170 |
| *NR3C1*-16 CpG 5 | 286806197 |
| *NR3C1*-16 CpG 6.7 | 286806208 |
|  | 286806210 |
| *NR3C1*-16 CpG 8 | 286806222 |
| *NR3C1*-16 CpG 9 | 286806240 |
| *NR3C1*-16 CpG 10 | 286806263 |
| *NR3C1*-16 CpG 11 | 286806273 |
| *NR3C1*-16 CpG 12 | 286806290 |
| *NR3C1*-16 CpG 13 | 286806350 |

**Table S2. The association of baseline academic pressure with subsequent anxiety symptoms.**

|  | **Anxiety symptoms status^a^** | | | | |  | **GAD-7 scores at follow-up^b^** | | | | |
| --- | --- | --- | --- | --- | --- | --- | --- | --- | --- | --- | --- |
|  | **Model 1** | |  | **Model 2** | |  | **Model 3** | |  | **Model 4** | |
|  | **OR (95% CI)** | ***P*-value** |  | **OR (95% CI)** | ***P*-value** |  | ***β* estimate (95% CI)** | ***P*-value** |  | ***β* estimate (95% CI)** | ***P*-value** |
| **Academic pressure at baseline** |  |  |  |  |  |  |  |  |  |  |  |
| Mild | 1.00 (reference) |  |  | 1.00 (reference) |  |  | Reference |  |  | Reference |  |
| Moderate | 8.33 (1.03~67.71) | 0.047 |  | 8.55 (1.05~69.54) | 0.045 |  | 3.44 (0.68~6.21) | 0.015 |  | 2.14 (-0.60~4.87) | 0.126 |
| Heavy | 25.00 (3.21~194.48) | 0.002 |  | 24.31 (3.12~189.22) | 0.002 |  | 8.25 (5.62~10.89) | <0.001 |  | 6.24 (3.48~9.01) | <0.001 |

Abbreviations: OR, odds ratio; 95% CI, 95% confidence interval; GAD-7, The Generalized Anxiety Disorder Scale-7.

a: logistic regression models were performed. Students with a GAD score≥10 at baseline and follow-up were considered as the case group (with persistently moderate to severe anxiety symptoms), and those with a GAD score <5 at baseline and follow-up were considered as the controls continuously not having anxiety symptoms. The reference group was the control group.

b: generalized linear regression models were performed.

Model 1: unadjusted model.

Model 2: adjusting for age, sex, BMI, cortisol, living, classmate relations, and teacher-classmate relations.

Model 3: unadjusted model.

Model 4: adjusting for age, sex, BMI, cortisol, living, classmate relations, teacher-classmate relations, and baseline anxiety symptoms scores.

| **Table S3. The mediating effect of *NR3C1* methylation in the association of academic pressure with anxiety symptoms.** | | |
| --- | --- | --- |
| **Mediating variable** | **Academic pressure (total effect)** | **Indirect effect^*^** |
|  | ***β* estimate (95% CI)** | ***β* estimate (95% CI)** |
| NR3C1-10 CpG 1 | 1.36 (0.74~1.98) | -0.01 (-0.12~0.02) |
| NR3C1-10 CpG 2 | 1.32 (0.71~1.93) | -0.01 (-0.01~0.03) |
| NR3C1-10 CpG 3.4.5 | 1.34 (0.73~1.95) | -0.001 (-0.07~0.04) |
| NR3C1-10 CpG 6.7.8 | 1.34 (0.73~1.95) | 0.01 (-0.02~0.11) |
| NR3C1-10 CpG 9.10.11 | 1.34 (0.73~1.95) | -0.001 (-0.06~0.02) |
| NR3C1-10 CpG 12.13 | 1.33 (0.72~1.95) | 0.01 (-0.02~0.11) |
| NR3C1-10 CpG 14 | NA | NA |
| NR3C1-10 CpG 15.16.17 | 1.32 (0.71~1.93) | -0.002 (-0.07~0.03) |
| NR3C1-10 CpG 18.19.20 | 1.32 (0.71~1.93) | 0.0003 (-0.04~0.05) |
| NR3C1-10 CpG 21 | 1.31 (0.70~1.92) | 0.01 (-0.02~0.18) |
| NR3C1-10 CpG 22.23.24 | 1.31 (0.70~1.92) | 0.004 (-0.02~0.18) |
| NR3C1-10 CpG 25.26 | 1.32 (0.71~1.93) | 0.0004 (-0.05~0.06) |
| NR3C1-10 CpG 27.28 | 1.32 (0.71~1.93) | 0.0004 (-0.04~0.07) |
| NR3C1-10 CpG 29 | 1.34 (0.72~1.95) | 0.001 (-0.06~0.08) |
| NR3C1-16 CpG 1 | 1.34 (0.72~1.96) | 0.02 (-0.02~0.16) |
| NR3C1-16 CpG 2 | 10.55 (-14.88~36.00) | 0.61 (-6.01~7.69) |
| NR3C1-16 CpG 3 | 1.33 (0.72~1.95) | 0.0001 (-0.08~0.07) |
| NR3C1-16 CpG 4 | 1.32 (0.69~1.95) | 0.03 (-0.02~0.16) |
| NR3C1-16 CpG 5 | 1.35 (0.73~1.96) | 0.002 (-0.03~0.07) |
| NR3C1-16 CpG 6.7 | 1.36 (0.74~1.97) | -0.03 (-0.18~0.03) |
| NR3C1-16 CpG 8 | 1.33 (0.72~1.95) | -0.004 (-0.10~0.03) |
| NR3C1-16 CpG 9 | 1.33 (0.71~1.95) | 0.02 (-0.03~0.15) |
| NR3C1-16 CpG 10 | 1.26 (0.62~1.90) | **0.17 (0.04~0.40)** |
| NR3C1-16 CpG 11 | 1.33 (0.71~1.94) | 0.0001 (-0.06~0.06) |
| NR3C1-16 CpG 12 | 1.31 (0.69~1.92) | 0.005 (-0.03~0.11) |
| NR3C1-16 CpG 13 | 1.36 (0.74~1.99) | -0.02 (-0.15~0.02) |

Abbreviations: 95% CI, 95% confidence interval; NA, not applicable or not available.

*: The indirect effect that is statistically significant is shown in bold type.
